# Effectiveness of the Combination of Probiotic Supplementation on Motor Symptoms and Constipation in Parkinson’s Disease

**DOI:** 10.7759/cureus.49320

**Published:** 2023-11-23

**Authors:** Athanasia Alexoudi, Lydia Kesidou, Stylianos Gatzonis, Christos Charalampopoulos, Areti Tsoga

**Affiliations:** 1 Department of Neurosurgery, National and Kapodistrian University of Athens, Evangelismos Hospital, Athens, GRC; 2 Neurology, Neurological Institute of Athens (NIA), Athens, GRC; 3 Dentistry, Santorini General Hospital, Santorini, GRC; 4 Internal Medicine, Hellenic Police Central Outpatient Care, Athens, GRC; 5 Department of Public Health Policy, University of West Attica, Athens, GRC

**Keywords:** neuroinflammation, probiotics, 25(oh)d3: 25 hydroxy vitamin d3, crocus sativus l, butyrate triglyceride, parkinson’s disease (pd)

## Abstract

Background: Parkinson’s disease (PD) reflects the second most common neurodegenerative disorder and is associated with high morbidity and mortality. Besides the motor features, non-motor symptoms, such as constipation, are very common. There is accumulating evidence that neuroinflammation is associated with the PD pathological processes. Alterations of gut microbiota and microbial metabolites have been linked to the pathogenesis of PD. Previous research has shown that probiotic supplementation has beneficial effects on motor and non-motor symptoms and especially on constipation.

Aim: In this study, we examine the effectiveness of a combination of probiotic supplementation (butyrate triglyceride 302.86 mg,* Crocus sativus L. *30 mg, and vitamin D3 100 mcg), on constipation and motor symptoms in PD.

Methods: The present study is a retrospective study that examined the existing medical records of patients with diagnosed PD, having chronic constipation and used the probiotic supplementation for its management. A total of 41 existing medical records were screened. Medical records were excluded in the case of participation in another study for PD, suffering from irritable bowel syndrome, organic constipation, long-term laxative use changes in the standard dopaminergic treatment, Mini-Mental Status Examination (MMSE) score<24, hospitalization and antibiotic medication, and diarrheal syndrome. Nine medical records were excluded, and a final number of 32 medical records was finally examined. All 32 patients had evaluations carried out at baseline and three months after supplement administration. A stool diary questionnaire, the Unified Parkinson's Disease Rating Scale III (UPDRS III), and the Schwab and England and the Hoehn and Yahr scales were used for the evaluation.

Results: The median defecation frequency was significantly improved. The supplementation administration significantly improved UPDRS III by 7.7% (from 35.72±15.51 to 32.97±15.71, p = 0.005) at month three, as compared to baseline. A positive effect was also seen in the Schwab and England scale. There was no effect on the Hoehn and Yahr scale.

Conclusion: The enteric microbiome composition is altered in PD, and there is accumulating evidence that probiotic supplementation could alleviate disease symptoms in neuroinflammatory disorders.

## Introduction

Parkinson’s disease (PD) reflects the second most common set of neurodegenerative disorder following Alzheimer’s disease (AD) and is associated with high morbidity and mortality (1.75-3.86 higher risk in PD) [1]. The aggregation and spread of α-synuclein (α-syn) is the pathological hallmark of PD, associated with disease severity and prognosis [2]. Clinically, PD is characterized by rigidity, bradykinesia, resting tremor, and postural instability [3]. Besides the motor features, non-motor symptoms are very common in patients with PD, including hyposmia, sleep disorders, depression, pain, sensory dysfunction, and constipation [4].

Levodopa remains the cornerstone for the treatment of PD. In the last decades, new agents have been introduced (dopamine agonists, monoamine oxidase type (MAO) B inhibitors, catechol-O-methyltransferase (COMT) inhibitors toward enhancing the dopaminergic load [5]. There is accumulating evidence that neuroinflammation is associated with the PD pathological processes [6].

The observed expression of α-syn in the central nervous system (CNS) and enteric nervous system (ENS) gave the hypothesis over the past two decades that ENS involvement may also be critical in the initiation and spread of PD. This brain-gut axis hypothesis has opened interesting perspectives in the pathogenesis of neurodegenerative diseases, especially PD [7]. It has been suggested that gut microbial toxins may induce the production of α-syn aggregates in the ENS, which can be subsequently propagated to and proliferated in the CNS in a prion-like manner through the vagus nerve [8]. The gut-brain axis is influenced by the gut microbiota, which is a diverse population of bacterial species [9]. Alterations of gut microbiota and microbial metabolites have been linked to the pathogenesis of PD according to several studies [10]. Previous research has shown that probiotic supplementation has beneficial effects on motor and non-motor symptoms and especially on constipation [11].

In this study, we examine the effectiveness of a combination of probiotic supplementation (butyrate triglyceride 302.86 mg, *Crocus sativus L.* 30 mg, and vitamin D3 100 mcg) on PD.

In patients with PD, the mechanism and contribution of butyric acid intake, in combination with probiotic and prebiotic agents, has been studied [12]. The role of short-chain fatty acids, especially butyric acid and all its products, has also been highlighted, and it is considered that on the one hand they protect neuronal cells from "death" and on the other hand they strengthen the mitochondria of neurons, giving the necessary power of action [13]. A number of research studies demonstrate the contribution of butyric acid and its other derivatives to the regulation of lipid metabolism and their therapeutic contribution not only in lipid disorders but also in PD [14,15].

*Crocus sativus L. *(*C. sativus*) or saffron is a well-known herb since ancient times, a member of the *Iridaceae *family, of the *Liliaceae *series. Its constituents include crocins, crocetin, picrocrocin, safranal, and flavonoids. Saffron and its components (crocin-1, crocin-2, and crocetin) may have therapeutic effects and prevent diseases in which there is abnormal aggregation of α-synuclein fibrils and amyloids, such as PD and AD [16]. The E46K mutation of the α-synuclein gene has been linked to autosomal dominant early-onset PD. E46K mutation has been shown to increase the pathogenicity of α-syn fibrils, enhance fibril formation, and promote higher levels of aggregation [17]. It appears that the effect of crocin inhibits and prevents the conversion of protofibrils into mature synuclein fibrils [18]. The beneficial effects in reducing depressive symptoms from the consumption of *C. sativus* extracts in patients with PD have been demonstrated and confirmed in experimental animal models [19]. Administration of saffron or its bioactive components has neuroprotective properties and lack of side effects or adverse events [20].

Vitamin D receptors are present in neurons and glial cells, with higher expression in the hippocampus, hypothalamus, thalamus, and gray matter [21]. Vitamin D contributes to neurotrophin regulation, neural differentiation, and maturation through its function of controlling the synthesis of growth factors (such as nerve growth factor) and the synthesis process of different neurotransmitters (such as acetylcholine, dopamine and gamma-aminobutyrate) [22]. The neuroprotective role of vitamin D in PD has been underlined in a study that suggests that vitamin D seems to inhibit the aggregation of α-synuclein [23]. In addition, the levels of vitamin D are inversely associated with the risk and severity of PD [24].

The aforementioned product is designed for the management of the non-motor symptoms of PD, and its efficacy is based on metabiotics that act on the gut-brain axis. In the present study, we want to examine its effects on constipation symptoms in PD. The secondary aim of the survey is to estimate the efficacy of the treatment in subpopulations of the patients with PD regarding their age, gender, years of disease, dose of dopaminergic medication, and possible change in motor symptoms and their daily life activities.

## Materials and methods

The present study is a retrospective study that examined the existing medical records of patients with diagnosed PD, having chronic constipation (based on the Rome III diagnostic criteria), and used the supplement containing butyrate triglyceride 302.86 mg, *Crocus sativus L.* 30 mg, and vitamin D3 100 mcg for the management of constipation for at least three months. The data were collected from the outpatient clinic of the Neurosurgery Department of the Evangelismos General Hospital of Athens, Greece, between January and December 2021. All the endpoints were part of our database. Included questionnaires and rating scales, which are also a part of our daily clinical practice, were filled at the beginning of supplementation and after completing the third month. Medical history and information concerning constipation symptoms were also recorded at the baseline assessment.

In our clinic, a stool diary questionnaire was included toward getting information of the changes of the defecation pattern. For the evaluation of the motor function, we used the Unified Parkinson's Disease Rating Scale III (UPDRS III). We also include the Schwab and England and the Hoehn and Yahr scales. Levodopa equivalent daily dose (LED) was calculated for each drug according to standard conversions [25].

**Figure 1:**
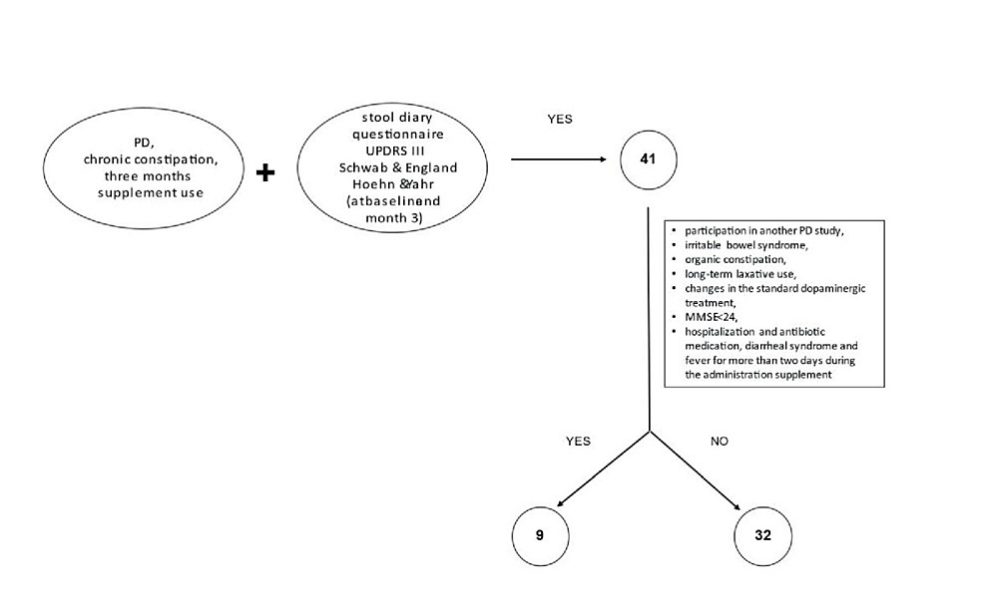
Retrospective study design

A total of 41 existing medical records were screened. All the patients were aged 18 and above. Medical records were excluded in the case of patient participation in another study for PD, suffering from irritable bowel syndrome, organic constipation, long-term laxative use (for more than seven days), changes in the standard dopaminergic treatment during the three-month period of the nutritional supplement use, Mini-Mental Status Examination (MMSE) score<24, hospitalization and antibiotic medication while taking the nutritional supplement, and diarrheal syndrome and fever for more than two days during the administration of the aforementioned food supplement. Considering the above excluding criteria, nine medical records were excluded, and a final number of 32 medical records was finally examined. All 32 patients had evaluations carried out at baseline and three months after supplement administration (Figure 1).

The data were coded, and therefore, identification of the subjects was not possible. The study was approved by the Ethical Committee of University of West Attica (IRB approval number: 46015-11/05/2022).

Statistical analysis

The results are expressed as means ± standard deviation (SD). The statistical processing of the data was carried out with the IBM SPSS Statistics for Windows, version 24 (released 2016; IBM Corp., Armonk, New York, United States). Chi-square test and t-test were used for comparisons (statistically significant when p < 0.05).

## Results

Among the participants, there were 18 females and 14 males with a mean age of 73.41±7.34 years. The mean years of disease were 9.47±7.34 years, and the mean levodopa equivalent dose (LED) they received was 690.13±379.51. We considered as a response the ≥1 point reduction in UPDRS III. All the patients were examined at their “best on.” Twenty-nine out of the 32 patients completed the three-month treatment with the combination of butyrate triglyceride/*Crocus sativus L.*/vitamin D3. Two patients discontinued the use of the supplement, and one developed transient rash.

At baseline, the median defecation frequency was 3.76 (two to six) times per week. During the three-month intervention, the median stool frequency increased to 5.41 (three to 10) times per week. Therefore, the improvement after the intervention was significant (p = 0.001). We also noticed significant changes regarding the sensation of incomplete evacuation, the recurrent abdominal pain, the quality of stools, and the number of bowel movements per week (p < 0.05).

The supplementation administration significantly improved Parkinsonian motor disability (UPDRS III) by 7.7% (from 35.72±15.51 to 32.97±15.71, p = 0.005) at month three, as compared to baseline (Table 1). Fifteen patients (51.72%) had a reduced score at the UPDRS III at month three, and we considered them as responders. Interestingly, all responders had a significant improvement in constipation (median defecation frequency increased from 3.86 to 6.64) compared with no responders (median defecation frequency increased from 3.67 to 4. 27). A positive effect was also seen in the Schwab and England scale (reduction 8.9%), reflecting an improvement in activities of daily life (Table 1). However, there was no effect on the Hoehn and Yahr scale (reduction 3.9%).

**Table 1:** Median defecation frequency (days per week), UPDRS III, Hoehn and Yahr, Scwhab and England scale scores during the three-month follow-up. UPDRS III: Unified Parkinson's Disease Rating Scale III

Endpoints	Baseline	Three months	p
Median defecation frequency	3.76 ±1.35	5.41±1.7	0.001
UPDRS III	35.72±15.51	32.97±15.71 (-7.7%)	0.005
Hoehn and Yahr scale	3.28±0.90	3.41±1.49 (3.9%)	0.442
Scwhab and England scale	58.28±18.14	63.45±20,23 (8.9%)	0.002

In addition, the responding and non-responding groups were further analyzed regarding gender, age (<75 years and ≥75 years), disease duration (<10 years and ≥10 years), and LED (≤600 and >600). Among the responders, we identified six females and nine males. Ten out of 15 were younger than 75 years old, and 11 had less than 10 years' disease duration. Only five of them received medication less than 600 LED. Thus, we found only minor differences, but not statistically significant (Table 2).

**Table 2:** Number of treated patients and their response to treatment with regard to a) gender, b) age, c) disease duration, d) LED. LED: levodopa equivalent dose

Subgroups		Responders	Non-responders	p
Gender	Female	6	10	0.09
	Male	9	4	
Age (years)	<75	10	7	0.59
	≥75	5	7	
Disease duration (years)	<10	11	7	0.36
	≥10	4	7	
LED	≤600	5	7	0.59
	>600	10	7	

Finally, a negative correlation was found between age and the Schwab and England scale (r = -0.331; level of significance of the correlation was set at p < 0.05), which was an expected finding, irrelevant to treatment supplementation.

## Discussion

The dietary supplement formulation given to the study patients contained butyric acid in the form of triglycerides, saffron extract, and vitamin D3. The results of our study showed that the patients who received the nutritional supplement, alongside their medication, noted a clear improvement in constipation and the motor symptoms of the disease.

The Schwab and England and UPDRS III scales showed differences before and after the administration of the nutritional preparation, with a strong indication of improvement of motor performance and activities of daily living, which could be partially interpreted as amelioration of the quality of life of the patients taking it.

Short-chain fatty acids, such as butyric acid, play a significant role in the intracellular energy metabolism. The protective effect of butyric acid in the development and progression of PD has been demonstrated in a number of in vivo and in vitro studies. Consistent with our results are the findings of Liu et al., who demonstrated an improvement of motor performance alongside with less dopaminergic degeneration in substantia nigra and striatum in a mouse model of PD, treated with sodium butyrate. The authors correlated the beneficial outcome with the increased colonic glucagon-like peptide-1 (GLP-1) level and upregulation of brain GLP-1R expression [26]. In addition, gene expression analysis in human mesencephalic cells overexpressing the αSyn treatment with butyric acid rescued aSyn-induced DNA damage [27].

The neuroprotective efficacy of saffron supplementation has been proven in animal models of PD. The possible mechanisms of action of saffron through antioxidant effects include reduction of endogenous levels of oxidative markers and pro-inflammatory cytokines often along with T cell activation and upregulating antioxidant enzymes, such as thioredoxin reductase (TR), glutathione-S-transferase (GST), and superoxide dismutase (SOD) [28].

Improvement in motor performance in PD patients has been demonstrated when vitamin D was added in their diet [29]. Nevertheless, it seems that there is no difference in clinical status and motor control when vitamin D supplement is used alone [24].

On the contrary, based on the Hoehn and Yahr scale, no change was observed after the administration of the aforementioned combination of probiotic supplementation. An interpretation of the above finding is that the Hoehn and Yahr scale is more heavily weighted toward some aspects of the disease, such as postural instability and does not necessarily reflect therapy‐related improvements in many other aspects of the disease, especially non-motor symptoms. The subsequent statistical analysis of the different groups of patients regarding gender, age, disease duration, and LED and their motor response to treatment did not reveal significant differences.

The probiotic supplementation on movement and metabolic parameters in people with PD has been assessed in several studies. Most of these agents have been approved as food preservatives in humans, and therefore, clinical application can be easily performed. Most of the studies demonstrated the favorable effects of probiotics on clinical performance and quality of life of people suffering from degenerative diseases, such as PD. However, the results are not homogenous. There is evidence that a short-chain fatty acid, propionic acid, induces gliosis, and neuroinflammation [30]. The presence of a variety of supplementary agents, the different disease models, and the absence of a standard dose application of the supplements are the main reasons for these conflicting results. Nevertheless, we must emphasize once again that the action of the components of the nutritional supplement work therapeutically through the gut microflora in the motor and non-motor symptoms of PD. Translational research results and optimal clinical protocols regarding the type and the concentration of supplements are needed toward improving our understanding and ameliorating the quality of life of patients with neuroinflammatory/neurodegenerative diseases.

Limitations of the study

Our study lasted for a very limited period. The small sample and the absence of control group are additional limitations. A barrier to enrolment was social distancing, which has proven a problem for many researchers during the COVID-19 pandemic. Apparently, these limitations have influence on the results. Prolonged supplementation treatment could become more effective, and larger population recruitment might provide more robust results. Moreover, we might notice a significant increase in the Hoehn and Yahr scale reflecting a change of the disease stage.

## Conclusions

This pilot study suggests that supplement formulation given to PD patients containing butyric acid in the form of triglycerides, saffron extract, and vitamin D3 is effective in increasing stool frequency and improving motor function and activities of daily living in the majority of the study population. Therefore, probiotic supplementation may be used as an complementary treatment for alleviating disease symptoms. The need for future research with a more robust design should be underlined.
